# A Revised Approach to Advance Personal Planning: The Role of Theory in Achieving “The Good Result”

**DOI:** 10.1007/s11673-023-10263-6

**Published:** 2023-05-30

**Authors:** Briony Johnston

**Affiliations:** University of Technology Sydney, Faculty of Law, PO Box 123, Broadway, NSW 2007 Australia

**Keywords:** Advance personal planning, Lawyers, Healthcare providers, Theoretical perspectives, Preventive Law Theory, Therapeutic Jurisprudence

## Abstract

This article explores traditional views of advance care planning in the broader context of advance personal planning, which also accounts for legal and financial matters. Criticisms of existing processes are noted, while the significance of interprofessional collaboration is highlighted. Reframing the purpose of advance personal planning as planning for the rest of life, rather than the end-of-life, and adopting a more holistic perspective informed by theory may help individuals to view advance personal planning as a routine, preventative exercise that safeguards their autonomy and well-being. Both lawyers and healthcare providers have an important role to play in reframing the purpose of advance personal planning. This revised approach is underpinned by the unification of two separate theoretical lenses: Preventive Law Theory and Therapeutic Jurisprudence. This combination enhances our understanding of what it means for people to truly achieve “the good result” (Holtz 2017) when planning ahead for their future legal, financial, health, and personal interests. Preventive Law Theory encourages an ongoing, collaborative relationship between lawyers and their clients, or healthcare providers and their patients, while Therapeutic Jurisprudence ensures an ethical approach to advance personal planning that accounts for all aspects of the individual’s well-being, including consideration of vulnerability, autonomy, and empowerment.

## Introduction

Advance personal planning (APP) provides a means of protecting individuals’ future legal, financial, health, and personal interests at a time when they can no longer speak for themselves (Waller et al. 2018). It is broader than advance care planning (ACP), which is confined to healthcare considerations only, and describes the process of a patient and their healthcare team discussing relevant options and potentially recording their preferences for treatment and goals of care (Sudore et al. 2017). This may produce a legal document known as an Advance Care Directive (ACD) or living will (Kimberly Buck et al. 2021; Baron 2019). APP is an umbrella term that encompasses planning ahead for health, legal, financial, and other personal matters so that wishes can be known and recorded ahead of a period of future incapacity or death (Waller et al. 2018). As opposed to being a distinct area of planning, APP simply refers to the full range of planning mechanisms that are available, such as: a Will, for disposing of property and assets following death; the appointment of an Enduring Power of Attorney, which authorizes another person to make decisions regarding an individual’s finances and property if they were experiencing a period of incapacity; the appointment of an Enduring Guardian, which enables someone to make decisions in relation to the future health or personal care that may be provided in the event the person cannot make those decisions for themselves; and documenting an ACD, which sets out a person’s wishes, values, and preferences for future medical treatment and personal matters, including end-of-life care.

Uptake of relevant instruments remains low and variable (Kimberly Buck et al. 2021; Sellars et al. 2021; Baron 2019). People are often hesitant to engage in APP, equating it with the end-of-life (Batchelor et al. 2019; Moore et al. 2019). The vast majority of existing literature has only focused on ACP processes, relating to the contemplation, discussion, and possible documentation of a person’s wishes relating to future healthcare and medical treatment only (Buck et al. 2019; Sudore et al. 2017) rather than considering the full range of advance planning mechanisms that are available. Recent literature has also questioned the utility of ACP, and whether it can in fact achieve its objective of providing high-value, goal-concordant care (Morrison, Meier, and Arnold 2021; McMahan, Tellez, and Sudore 2021; Morrison 2020).

Reframing the purpose of APP as planning for the rest-of-life (Masters, Wylie, and Hubner 2021; Yapp et al. 2018) and adopting a more holistic perspective informed by interdisciplinary collaboration and the application of theory may help individuals to perceive APP as a routine, preventative exercise that safeguards a person’s autonomy and well-being (Yapp et al. 2018; Brinkman-Stoppelenburg, Rietjens, and van der Heide 2014; Rhee, Zwar, and Kemp 2012). This revised approach is founded upon the unification of two separate theoretical perspectives: Preventive Law Theory; and Therapeutic Jurisprudence. The combination of Preventive Law Theory and Therapeutic Jurisprudence, termed “theralaw” (Barclift 2008, 36), enhances our understanding of what Holtz (2017) has termed “the good result.” Preventive Law Theory encourages an ongoing, collaborative relationship between professionals and their individual clients or patients, while Therapeutic Jurisprudence ensures an ethical approach to APP that accounts for all aspects of an individual’s well-being, including consideration of vulnerability, autonomy, and empowerment in the process of planning ahead.

## Advance Personal Planning

APP is concerned with an individual’s wishes, values, and preferences associated with their money, property, legal affairs, and medical care at a time in the future when they may be either too ill to speak for themselves or cannot communicate their decisions due to a decline in, or loss of, capacity (Sinclair et al. 2020; Detering et al. 2019; NSW Health 2018; Waller et al. 2018). APP may involve a series of acts, including thinking about preferences for the future, discussing these wishes with important parties including family, appointed decision-makers and professionals, and potentially taking the step of formally recording these intentions (Department of Health 2021; Sudore et al. 2017). Relevant instruments include: a Will; the creation of an ACD; an Enduring Power of Attorney appointment; and an Enduring Guardian appointment. APP has been described as empowering (Baron 2019; Ries et al. 2018; Yapp et al. 2018) and an important exercise in self-determination (Close et al. 2021; Sinclair et al. 2021; Moore et al. 2019) as it represents “a means of extending the autonomy of patients to stages in life where they have become incompetent” (Brinkman-Stoppelenburg, Rietjens, and van der Heide 2014, 1000). However, uptake of APP remains low and variable (Buck et al. 2021; Sellars et al. 2021; Baron 2019) and the final steps of maintenance or ongoing reflection are often overlooked (Buck et al. 2021; Baron 2019; Buck et al. 2019; Scott et al. 2013).

### Criticisms of Current Processes

While discussions of APP are only now emerging in the available literature (Waller et al. 2018), the utility and success of ACP processes have recently been questioned (Orsatti 2022; Morrison, Meier, and Arnold 2021; McMahan, Tellez, and Sudore 2021; Morrison 2020). Morrison et al. argue that existing evidence reveals ACP “fails to improve end-of-life care” and the objectives of delivering goal-concordant care and improving quality of life for patients are not being met (Morrison, Meier, and Arnold 2021, 1575). Similarly, appointed decision-makers are often unable to meet their obligations, as they are either not aware of the person’s preferences (Morrison 2020, 878) or cannot enact their wishes in a meaningful way in complex clinical environments (Morrison, Meier, and Arnold 2021, 1575–1576). Similarly, even if an ACD has been prepared, it may not be accessible at the point of care, or instructions provided may be either too broad or too specific to be applied in the treatment context (Morrison, Meier, and Arnold 2021, 1576). Communication is another significant factor, as patients are often not talking about their wishes with their healthcare provider or other members of their support community, including family, carers, or appointed decision-makers (Morrison 2020, 878). From a professional perspective, clinicians may not have time to engage in planning conversations or may not feel adequately equipped to introduce the topic with their patients (Morrison 2020, 878).

### The Need for Interdisciplinary Collaboration

The traditional approach towards ACP has prioritized communication and shared decision-making between healthcare practitioners and patients (Sudore et al. 2017; Rhee, Zwar, and Kemp 2012). However, evidence indicates people may be more inclined to speak about their healthcare wishes with lawyers as part of the broader exercise of planning for the future (Orsatti 2022, 157; Masters, Wylie, and Hubner 2021; Rolnick et al. 2019). Interestingly Rolnick et al. found that, of the eleven participants interviewed regarding their experience of creating an ACD, six had prepared the document in consultation with a lawyer as it was often incorporated as part of the estate planning process (Rolnick et al. 2019, 3–4). Even the four participants who completed their ACD through a health institution had not sought the involvement of health professionals when documenting their wishes. Participants reported that they appreciated lawyers’ knowledge of, and attention to, legal details which gave them a sense of assurance that their wishes would be followed in future (Rolnick et al. 2019, 3–4). This gives people confidence that their plans for the rest-of-life are valid, and their future goals and preferences will be safeguarded.

Masters et al. also highlighted the role of lawyers in assisting individuals to complete APP (Masters, Wylie, and Hubner 2021). In normalizing discussions about future wishes, especially at a time when individuals are in good health, we need to recognize the vital role of lawyers in initiating these conversations. While respondents were most likely to speak with their spouse/partner or a family member about their end-of-life wishes, they were more likely to speak with a lawyer regarding APP than a healthcare professional (Masters, Wylie, and Hubner 2021, 12). This highlights a key area in which lawyers can play a continuing role in APP, ensuring plans are accurate and accommodate all aspects of an individual’s welfare when planning for the rest-of-life.

This dichotomy creates tension, as lawyers are not perceived as having the requisite medical knowledge to prepare robust documents relating to future healthcare and treatment (Orsatti 2022, 157). However, Orsatti highlights the benefit of involving lawyers in ACP processes, as they are able to assist people to select “the healthcare agent best suited to the task of promoting the client’s wishes,” ensure preferences are shared widely among an individual’s support community, including with their family, healthcare provider, and appointed decision-makers, and ensure ACP instruments “comply with applicable law to protect clients against the possibility of the documents being ineffective when needed most” (Orsatti 2022, 158). Given that lawyers may be assisting individuals with other planning instruments, including the preparation of a Will or the appointment of a decision-maker, they are likely to have a full overview of the person’s circumstances, including who would be best placed to support them in future. Further, lawyers can provide resources for decision-makers that can help explain the purpose of their role, what they would be required to do, and when their powers would begin to operate.

While the benefits of including lawyers are clear, attention should also be directed to lawyers’ attitudes towards engaging with people in these areas. Ries et al. conducted a survey of lawyers in Alberta and found the main barrier to assisting clients with ACP was the person’s “lack of preparedness to engage in ACP” and that nearly all respondents “said they never or seldom find ACP discussions upsetting or uncomfortable” (Ries et al. 2018, 691). However, just under half of the respondents “revealed some degree of concern with their own lack of knowledge about the medical aspects of ACP and health sector policies and practices” (Ries et al. 2018, 691). This discussion highlights that, while lawyers can fulfil a vital role in APP processes, including traditional ACP discussions, there is a need for enhanced interprofessional collaboration between the health and legal fields. In this way, people can be assisted to plan ahead holistically, benefiting from the expertise of both healthcare providers and legal practitioners, each bringing their unique insight to an individual’s circumstances.

Now that we have identified the need for, and benefits of, interprofessional collaboration in a reframed approach to APP, the relevance of theory will be explored in more detail.

## Preventive Law Theory

Preventive Law Theory was first articulated by Brown and Daure in the 1950s (Brown 1956) and developed further under Stolle in the 1990s (Stolle et al. 1997; Stolle and Wexler 1997; Stolle 1996). The introduction of “theralaw” (Barclift 2008, 36) also occurred at this time (Winick 2001). Essentially, Preventive Law Theory is a shift in focus from reacting retrospectively to a crisis and instead taking active steps to prevent it from occurring in the first place (Brown 1956; Goldblatt, Hardaway, and Scranton n.d.; Barton n.d.) through communication and planning (Winick 2001; Stolle 1996) rather than adopting a conflict-driven approach (Zirkel n.d.). These principles very much echo our existing, health-informed processes to ACP where communication and forward-planning are championed. It introduces the concept of “legal hygiene” (Zirkel n.d.) and the need for regular “legal checkups” (Winick 2001; Stolle et al. 1997; Brown n.d.; Wexler n.d.) which, similar to health checkups, are designed to provide a holistic view of the person’s life, including their values, finances, concerns, and family structure (Stolle et al. 1997). The goal is to protect their interests and proactively guard against future legal conflicts (Stolle et al. 1997; Stolle 1996). Prevention and protection are key goals of APP, just as in ACP, and continue to apply when reframing our approach as planning for the rest-of-life. The goal is still to prevent future conflicts or issues arising in future, while ultimately protecting the person’s wishes and preferences.

Preventive Law Theory ensures people are able to actively engage in the process and collaborate with their lawyer, something that is often missing in litigation (Barclift 2008). Lawyers work alongside clients, providing them with a deeper understanding of any particular issues that may arise in future (Barclift 2008; Stolle 1996), much like healthcare providers do when considering a particular diagnosis or disease trajectory. This early intervention helps to prevent legal risks from occurring or minimize their impact if they do eventuate (Barclift 2008). The preventive lawyer must understand the potential problem and devise appropriate solutions in connection with the person’s whole environment, acknowledging their values and well-being (Barton 2009). Transparency is key, as the lawyer needs to be provided with all of the relevant facts and the individual must be made aware of the applicable law that will operate in order for Preventive Law Theory to be effective (Brown 1956). This collaborative approach between lawyers and clients provides a clear direction when planning for the rest-of-life. It is vital to understand not only what is important to a person now but what their wishes and preferences are for the rest-of-life. Armed with this information, lawyers can assist clients to plan ahead in a way that acknowledges and incorporates their wishes and protects their interests in a meaningful way.

While many lawyers are undoubtedly implementing aspects of Preventive Law Theory, there is a desire to make the approach more systematic (Stolle and Wexler 1997). APP has a significant preventive role in protecting individuals if they were to experience future periods of incapacity. To ensure this protective role is realized, APP instruments need to be carefully prepared to minimize the risk that any legal planning tools “can be abused and transformed from a preventive tool to a harmful one” (Doron and Gal 2007, 51). This careful preparation can be undertaken by a lawyer, in conjunction with a health professional, with a clear understanding of a person’s goals and preferences when planning for the rest-of-life, preventing future issues, and guarding against opportunities for abuse or manipulation.

## Therapeutic Jurisprudence

As Therapeutic Jurisprudence aims to humanize the law and make it a more positive experience for those involved (Wexler n.d.), it can improve our approach to APP. Therapeutic Jurisprudence is concerned with the effect the law can have on the psychological well-being of individuals (Stolle and Wexler 1997), prompting us to work to increase therapeutic, and decrease antitherapeutic, consequences of APP (Glover 2011; Kapp 2009). Therapeutic Jurisprudence also introduces the concept of “psycholegal soft spots” (Winick 2001; Wexler n.d.). While “legal soft spots” relate to circumstances that might lead to future legal conflicts for the person, a concept which is highly relevant to Preventive Law Theory, “psycholegal soft spots” are concerned with the individual’s social connections and well-being, which should be taken into account when attempting to prevent conflict or considering which legal tools are best suited to the situation (Stolle et al. 1997). Our understanding of a person’s well-being should acknowledge the related concepts of vulnerability, autonomy, and empowerment.

### Vulnerability

Properly understood, vulnerability should be recognized as experiences “of chronic and episodic dependency across the lifecourse” (Fineman 2008, 11). Building on this understanding of vulnerability as periods of heighted dependency, our focus is directed to relationship-based and circumstantial elements that can make someone vulnerable (Hall 2009). It also individualizes the concept as it can manifest differently for every individual, if at all (Hall 2009). Vulnerability is highly contextual as opposed to comparative (Boni-Saenz 2019), meaning it should be considered at regular intervals throughout a person’s life, as it forms an essential foundation for future planning.

### Autonomy

The Australian Health Ministers’ Advisory Council states autonomy “is generally understood as a person’s ability to make self-determining choices and direct his or her own life” (Australian Health Ministers’ Advisory Council 2011, 17) which emphasizes the concepts of self-direction and control. Further, Abrams connects these concepts with the values of authenticity and self-identity (Abrams 1999). Self-direction enables an individual to engage in decision-making that is free from external influences (Abrams 1999). Authenticity is paramount to autonomy, as it ensures an individual “is not merely the mouthpiece of other persons or forces. Rather, his tastes, opinions, ideals, goals, values, and preferences are all authentically *his*” (Feinberg 1989, 32). Similarly, self-identity enables an autonomous person to not be “exhaustively defined by his relations to any particular other” (Feinberg 1989, 31–32). Finally, autonomy can be understood “as a kind of competency … that makes it possible to act in a self-aware and self-directed fashion” (Abrams 1999, 814–15). This conceptualization can allow for a deeper understanding that, if an individual lacks the ability to self-manage a specific area of their lives, such as their financial affairs, they may still retain the capacity, and autonomy, to make decisions regarding their healthcare, or vice versa. This is vital for individuals when planning for the rest-of-life, in the event their competency may change over time.

Relational autonomy is also promoted by Gray et al. (Gray et al. 2019). While autonomy in health contexts has traditionally been recognized as “the capacity to make decisions independently” (Gray et al. 2019, 80) this fails to account for the participation of people close to the individual in the decision-making process (Baron 2019; Gray et al. 2019) and the potential reliance that may be placed upon them, for example, if required to make a decision with respect to the treatment of another in light of a serious diagnosis (Gray et al. 2019). However, familial relationships or caregiver responsibilities can also present “ethically challenging” circumstances with regard to autonomous decision-making (Tobin Tyler 2019, 3). This requires a careful balance, acknowledging the significant role carers or family may play in the individual’s life while ensuring that the wishes being communicated are in fact those of the individual (Australian Guardianship and Administration Council 2019; Tobin Tyler 2019). Therefore, prioritizing a person-centred approach for healthcare or legal matters must take these considerations into account, particularly when planning for the rest-of-life.

### Empowerment

Karl has defined empowerment as “a process of awareness and capacity building leading to greater participation, to greater decision-making power and control” (Karl 1995, 14). The knowledge of the full range of available APP mechanisms, not simply those associated with ACP, would enable individuals to make a wider range of decisions and take control of their current affairs, while also retaining control through the relevant documents and appointments at a time when they may not be able to speak for themselves. Participation is key, but it must be meaningful, as “[e]mpowerment is demonstrated by the quality of people’s participation in the decisions and processes affecting their lives” (Oxaal and Baden 1997, 7).

Within the health promotion context, Mason et al. highlight that empowerment is largely concerned with enabling individuals to recognize and utilize their personal power, with an acknowledgement of others who are involved in any relevant processes (Mason, Backer, and Georges 1991). This perspective allows individuals to take control over their future while also considering and respecting others who are involved, including family, trusted individuals, and appointed decision-makers. It provides greater opportunities for people to exercise control over their own lives and affairs, allowing them to express their wishes and retain their voice when future decisions must be made. Reframing APP as planning for the rest-of-life promotes the ongoing decision-making authority of individuals. Both lawyers and healthcare providers have a key role to play with respect to knowledge provision to ensure people have both a greater level of confidence with, and awareness of, the full range of available planning mechanisms.

## “The Good Result”

Holtz’ expression of “the good result” is essential to understanding how APP could be informed by a more ethical and holistic perspective (Holtz 2017). While lawyers may lack training relating to the traditional focus of Therapeutic Jurisprudence, being the psychological well-being of clients (Stolle et al. 1997), legal practitioners are readily able to collaborate with individuals and their care providers to consider what a “good result” would be in light of their personal circumstances. As such, “the good result” must encompass legal, financial, social, and personal well-being, including relevant health matters. A shift from the traditional legal transaction approach to a more communicative and collaborative process is vital, as “the good result” “is achieved through time, patience and perseverance … practitioners must be continually ready and willing to provide their clients insight and guidance as they make a variety of life-impacting decisions” (Holtz 2017, 86–87). Further, consistency and continued consideration of a person’s subjective circumstances, rather than a one-off consultation, is needed to produce the best outcome for the individual (Holtz 2017), both currently and into the future. This informs our ethical understanding of the possibility of reframing APP as planning for the rest-of-life, as “the good result” may change depending on a person’s stage of life. Lawyers and doctors, along with their clients and patients, must continue to work together over time, engaging in ongoing communication, collaboration, and reflection, to ensure their goals continue to be achieved.

Our understanding of vulnerability as flexible periods of heightened dependency is informed by the theoretical framework and our understanding of “the good result.” Professionals can act to reduce opportunities for harm that may come from circumstances where an individual is relying on others for support or is otherwise dependent. This is overtly linked to Preventive Law Theory and the ability of lawyers to assist clients in protecting themselves. They may put mechanisms in place that will help prevent legal conflicts from arising in future or may offer protection for clients from people in their lives who may be actively placing them in a vulnerable position. Therapeutic Jurisprudence then comes into play as both health and legal practitioners will need to be mindful of the person’s well-being, including legal, social, financial, and personal factors, when determining the best course of prevention or protection, both currently and when planning for the rest-of-life.

Autonomy, informed by authenticity, ensures decisions that people make are true to, and clearly represent, their values, wishes and preferences, and not those of their advisors or those close to them. Self-direction is also key to achieving “the good result,” as decisions must be made according to the personal goals and values that are prioritized by an individual. Partial or limited autonomy also accounts for instances where, for example, a person may have competence in one area of their life, such as their healthcare, but may need assistance through APP mechanisms to manage their finances. The role and significance of personal relationships should also be recognized, as they are an important factor when considering the well-being of individuals, in line with Therapeutic Jurisprudence. Further, under Preventive Law Theory, relationships must be acknowledged when determining how best to help people guard against future conflicts or proposed risks.

With respect to empowerment and its connection to “the good result,” Holtz draws on key aspects of planning for the rest-of-life in the following analysis:

… [t]he lure of contemporary empowerment has encouraged individuals to ‘avoid’ probate, adopt alternate methods of wealth transfer, and ‘secure’ their own future. Once a ‘spectator’ in the estate and financial planning process, individuals are encouraged to manage their own destiny. (Holtz 2017, 83)

The concept of self-management is again emphasized and clearly promotes the principles of Preventive Law Theory outlined above. Holtz’ conceptualization also highlights the significance of delegation, as people engaging in APP processes are able to anticipate and consider delegating future decision-making powers to trusted individuals (Holtz 2017). This echoes the earlier significance of control, as APP not only allows for current consideration of an individual’s affairs but what they would like to happen in future and who they would like to act on their behalf. This ensures APP, as planning for the rest-of-life, achieves “the good result” not only during the planning process itself, but in the future as well.

## A New Approach

APP would clearly benefit from a redirection under theralaw (Kapp 2009) with a focus on planning for the rest-of-life. Unifying the perspectives of Preventive Law Theory and Therapeutic Jurisprudence creates a more beneficial model than if either of them were adopted in isolation, as they strengthen one another when used together (Winick 2001; Stolle 1996). While the principles of Therapeutic Jurisprudence are significant, they can only be operationalized successfully by implementing Preventive Law Theory tools (Winick 2001; Stolle 1996) such as regular “legal checkups.” Similarly, by adopting a practice that is grounded in Preventive Law Theory, professionals will find themselves able to respond more easily to therapeutic concerns (Stolle 1996) and actively understand and protect the well-being of their clients and patients. The combination of Preventive Law Theory and Therapeutic Jurisprudence enhances their individual flexibility, so they can be introduced across a greater range of professions. Therapeutic Jurisprudence also represents an important tool via which we can measure improvements to the quality of life of individuals (Kapp 2009) with particular reference to their well-being. Wexler highlights the interaction between the two, as Preventive Law Theory seeks to guard against “legal soft spots” and Therapeutic Jurisprudence helps identify any “psycholegal soft spots” that, while not recognized as significant legal issues, may nevertheless have a real impact on a person’s well-being (Wexler n.d.). As Therapeutic Jurisprudence “is far from self-executing, and is in desperate need of a facilitating legal structure or context,” (Stolle and Wexler 1997, 27) Preventive Law Theory provides the necessary foundation for Therapeutic Jurisprudence to be introduced and implemented with confidence. The practicality of Preventive Law Theory is thus supplemented by the ethical framework offered by Therapeutic Jurisprudence (Stolle and Wexler 1997; Wexler n.d.).

Within the context of APP, lawyers play an important role in assisting clients to control their own affairs and to put steps in place for their management in the event they are not able to act autonomously in future. Similarly, healthcare providers have vital insight with respect to the health of their patients, including any recent diagnosis or potential disease trajectories. Both professions must consider the person’s well-being (Stolle 1996), including what is important to them and what they wish to prepare for or guard against in future. Lawyers must assist their clients to understand the range of legal protections available to them and demonstrate how APP can be used to record and protect the person’s values, wishes, and future intentions when they are managing their affairs, in line with Preventive Law Theory. Similarly, healthcare providers can assist with this sharing of knowledge, and encourage patients to think about their preferences for future treatment.

Clearer links should be made to the therapeutic nature of APP and the vital role well-being plays in achieving “the good result.” Professionals working in this area must actively engage with their clients’ and patients’ needs, values, and wishes across health, lifestyle, and social sectors, accounting for a broader and more ethical approach according to Therapeutic Jurisprudence. Using the example of ACDs, a document with which both professions are familiar, Ellison highlights the concepts of participation and control. An ACD allows an individual to “maintain control over their medical treatment” and aims “to uphold principles of autonomy and self-determination for people with diminishing capacity” (Ellison et al. 2004, 156), as well as allowing for increased participation in future processes. Expanding the focus of autonomy provides a broader framework for appointed decision-makers when called upon to act on behalf of an individual, as they will have a greater understanding of the context in which the wishes were recorded (Yapp et al. 2018). This provides an ethical, holistic view of a person’s life and their personal framework when planning for the rest-of-life.

Consideration of a wide range of barriers encourages a more comprehensive view of an individual’s circumstances, which is very important when considering both their legal hygiene under Preventive Law Theory and their well-being in accordance with Therapeutic Jurisprudence. Therapeutic Jurisprudence has a clear goal within the field of APP to encourage a holistic assessment of the impact legal and planning processes can have on people and those around them, as any legal interactions can have consequences on the well-being of all involved (Kapp 2009). APP has both therapeutic and antitherapeutic effects. Positive consequences include personal freedom in creating plans and personal connection with, and support from, professionals during the process (Glover 2011) including lawyers and healthcare providers. However, negative consequences such as anxiety associated with mortality and the potential loss of capacity (Glover 2011), conflicts within families (Barry 2017), and concerns about the duration and cost of planning processes (Close et al. 2021; Sellars et al. 2019) must also be acknowledged.

With respect to empowerment, facilitating individual participation when planning for the rest-of-life may help to guard against legal conflicts or prevent adverse medical events from arising in future. Further, it may help to reduce antitherapeutic effects that a person may otherwise experience if they were not aware of the available legal protections and their purpose. The element of control is also satisfied with respect to APP generally. The process of planning for the rest-of-life allows people to not only record their wishes, values, and preferences, but also retain control over the direction of their remaining years. They are able to appoint people to whom they delegate decision-making responsibility and specify their preference for healthcare to guide future decisions at a period when they may be experiencing incapacity. The key role that lawyers and healthcare providers play in the promotion and completion of APP instruments can help safeguard individuals in circumstances where people close to them may be either pressuring them to make certain arrangements or exercising undue influence for their own personal motives (Lewis 2018; Holtz 2017). Adopting this holistic view of the person’s life and well-being also links to Therapeutic Jurisprudence, as “the good result” for each individual needs to be informed by their personal circumstances and preferences.

Prioritizing communication enhances an individual’s participation and continued involvement in managing their affairs (Department of Health 2021, 19; Rodi et al. 2021). Engaging in the process of APP can be empowering for individuals (Glover 2011; Kohn 2006) as long as they are provided with opportunities for meaningful participation (Purser and Sullivan 2019; Yapp et al. 2018, 138–39) and are fully informed about the available planning mechanisms and their intended purpose (Kapp 2009; Doron and Gal 2007). Preventive Law Theory helps subvert the power imbalances that can exist in lawyer–client relationships by encouraging collaboration and the client’s active involvement (Barton n.d.), proceeding from a perspective of empowerment rather than vulnerability. Communication and participation are vital, as they preserve the person’s autonomy and control over their own affairs (Kohn 2006) and have the potential to decrease antitherapeutic consequences that may otherwise arise. By adopting this new approach to APP, involving a holistic examination of a person’s life and encouraging collaboration that accounts for any vulnerability, while upholding individual autonomy and promoting opportunities for empowerment, lawyers and healthcare providers are able to work with the individual to identify and achieve their “good result.”

Beyond the initial creation of APP instruments, establishing an ongoing plan for consultation and review of prepared mechanisms also increases the opportunity for therapeutic interactions and enhances the relationship between professionals and their clients or patients (Stolle et al. 1997; Stolle 1996). These regular “legal checkups” are encouraged under Preventive Law Theory, as well as being a regular part of routine healthcare practice. This proactive approach has a dual purpose of lowering both the chance and associated costs of future conflicts, while encouraging people to engage in all available forms of advance planning, which in turn produces therapeutic benefits (Stolle 1996). Interdisciplinary collaborations have been lacking in the field of APP despite the overt social aspects of legal interactions, including the mental well-being of clients (Stolle et al. 1997). The adoption of a holistic approach to APP goes beyond the skillset of the individual lawyer or healthcare provider (Orsatti 2022; Rolnick et al. 2019; Ries et al. 2018), highlighting the need to both consider and actively encourage collaboration between these professions (Heyland 2019; Johnson et al. 2016).

## Conclusion

While APP provides an important means for individuals to plan ahead regarding their future legal, financial, personal, and health affairs, uptake is low and too often associated only with the end-of-life. By reframing our approach to APP as planning for the rest-of-life and applying the unified theoretical perspectives of Preventive Law Theory and Therapeutic Jurisprudence under “theralaw,” we are paving the way for a new, interdisciplinary, ethical method of planning ahead that accounts not only for a person’s wishes but focuses on preventing conflict and protecting their well-being. Lawyers and healthcare providers both have a key role to play in this new approach, and should encourage an ongoing, collaborative relationship with those who are choosing to plan ahead for the rest-of-life.
